# (Pro)renin receptor aggravates myocardial pyroptosis in diabetic cardiomyopathy through AMPK-NLRP3 pathway

**DOI:** 10.3389/fphar.2024.1453647

**Published:** 2024-10-31

**Authors:** Shengnan Li, Jingjing Zhang, Yuewen Zhao, Li Kang, Haipeng Jie, Bo Dong

**Affiliations:** ^1^ Department of Cardiology, Shandong Provincial Hospital, Cheeloo College of Medicine, Shandong University, Jinan, China; ^2^ National Key Laboratory for Innovation and Transformation of Luobing Theory, The Key Laboratory of Cardiovascular Remodeling and Function Research, Chinese Ministry of Education, Chinese National Health Commission and Chinese Academy of Medical Sciences, Department of Cardiology, Qilu Hospital of Shandong University, Jinan, China; ^3^ Department of Cardiology, Shandong Provincial Hospital Affiliated to Shandong First Medical University, Jinan, China; ^4^ Division of Cellular and Systems Medicine, School of Medicine, University of Dundee, Dundee, Scotland, United Kingdom

**Keywords:** (pro)renin receptor, diabetic cardiomyopathy, pyroptosis, NLRP3, AMPK (AMP-activated protein kinase)

## Abstract

**Introduction:**

As one of the most common complications of diabetes, diabetic cardiomyopathy (DCM) is the main cause of heart failure in patients with diabetes. However, the lack of effective treatments for DCM remains a clinical challenge. (Pro) renin receptor (PRR) is a member of renin angiotensin aldosterone system (RAAS). Here, we aim to determine whether PRR is involved in myocardial pyroptosis in diabetic cardiomyopathy.

**Methods:**

We established diabetic rats model by intraperitoneal injection of streptozotocin (STZ). PRR overexpression adenovirus or PRR knockdown adenovirus was injected into the tail vein. Western blot, histopathology and immunohistochemistry staining, ELISA and Echocardiography were used to detect cardiac function changes and myocardial injury levels of DCM rats. Primary cardiomyocytes were stimulated with high glucose and PRR overexpression or PRR knockdown was achieved by adenovirus transfection, we also used the inhibitor of AMPK to decrease the activity of AMPK. Western blot, Real-time PCR, Immunofluorescence and ELISA were used to detect the level of PRR and pyroptosis in cardiomyocyte.

**Results:**

We found that high glucose increased the expression of PRR in heart. After overexpression of PRR, the expression of the pyroptosis related proteins such as Caspase-1, IL-1β, IL-18, and NLRP3 was significantly increased, the phosphorylation level of AMPK was significantly decreased, and the fibrosis level was significantly increased, thus aggravating the cardiac function injury of DCM. On the contrary, PRR knockdown can alleviate the level of myocardial pyroptosis in DCM and improve cardiac function. The related mechanism was that PRR could inhibit AMPK phosphorylation and promote the activation of NLRP3 inflammasome.

**Discussion:**

PRR aggravated pyroptosis of cardiomyocyte, increased the dysfunction of cardiomyocyte, and may be related to the decrease of AMPK phosphorylation and the overactivation of NLRP3. This may provide new ideas and targets for the treatment of DCM.

## 1 Introduction

It is reported that the number of diabetic patients is projected to reach about 642 million around the world by 2040 (Ogurtsova et al., 2017). As one of the leading complications of diabetes mellitus (DM), diabetic cardiomyopathy (DCM) is usually characterized by abnormal myocardial structure and function in diabetic patients without basic diseases such as hypertension or coronary heart diseases (Boudina and Abel, 2010). DCM usually increases the risk of heart failure and is a major cause of death in patients with chronic diabetes (Paolillo et al., 2019). However, there is still no effective medical treatment and its specific pathogenesis remains to be clarified.

In DCM, the death of terminally differentiated cardiomyocytes is a key molecular event in the progression of it, as it can lead to loss of contractile units and impaired systolic function (Frati et al., 2017). Pyroptosis is a newly discovered, pro-inflammatory programmed cell death, which is different from necrosis and apoptosis in mechanism (Bergsbaken et al., 2009). It mainly depends on cysteinyl aspartate-specific proteinase-1 (Caspase-1) and is closely related to the activation of NLRP3 inflammasome, which is a multiple protein complex composed of NLR, ASC and Caspase-1 (Shi et al., 2017; Gross et al., 2011). In diabetes, hyperglycemia can stimulate the activation of NLRP3, thus activate Caspase-1, which can promote the maturation of cytokines pro-IL-1β and pro-IL-18 to release the cleaved fragments of active IL-1β and IL-18, and lead to pyroptosis (Abderrazak et al., 2015). As the core of the whole response, NLRP3 and Caspase-1 are considered as markers and targets of pyroptosis in diabetic cardiomyopathy (Zhou et al., 2018). As a highly conserved cellular sensor, AMP-activated protein kinase (AMPK) plays a key role in regulating energy homeostasis and many cellular statuses (Meijer and Codogno, 2007). Interestingly, some studies indicate that the activation of AMPK can reduce the upregulation of NLRP3 inflammasome in some pathological processes including diabetes, pain, ischemic stroke and endoplasmic reticulum stress (Qiu et al., 2016; Bullon et al., 2016; Li et al., 2015).

As a member of the renin angiotensin aldosterone system (RAAS), (pro) renin receptor (PRR) is a protein with a molecular weight of 37–39 kDa, its activation promotes angiotensin I production. In addition, the binding of renin and prorenin triggers intracellular signaling pathways independent of Ang Ⅱ production, including the activation of RAAS-independent mitogen-activated protein kinase (MAPK) and its function as an accessary protein of vacuolar H^+^-ATP enzyme (V-ATPase) and as a component of Wnt/β-catenin signaling pathway (Ichihara and Yatabe, 2019). Interestingly, more and more studies have shown that PRR may not affect through the Ang II-dependent pathway. This suggests that PRR may be a potential target for the treatment of cardiovascular and renal complications.

Many studies have found that the expression of PRR is increased in the myocardium of diabetes, myocardial infarction and heart failure, and is closely related to its pathological changes (Connelly et al., 2011; Mahmud et al., 2014; Hirose et al., 2009). However, it is not clear whether PRR is involved in the pyroptosis of DCM and its specific regulation mechanism.

In this study, we studied the effects of PRR on cardiomyocytes treated with high glucose and on myocardial pyroptosis in rats with diabetic cardiomyopathy, and further explored the possible mechanism of PRR participating in AMPK/NLRP3. Our results provide a new insight into the role of PRR in DCM damage.

## 2 Material and method

### 2.1 Construction of adenovirus overexpressing PRR

We commissioned GenePharma (Shanghai, China) to design the amplified open reading frame (ORF) of the PRR gene and inserted it into the plasmid pDc316 to construct the pDc316-PRR shuttle plasmid. Then the pDC316-PRR plasmids containing MCMV promoter or pDC316-NC plasmids were co-transformed with adenovirus vectors to construct PRR-overexpressing recombinant adenovirus (Ad-PRR) and NC containing recombinant adenovirus (Ad-EGFP), in which Ad-EGFP was used as the control group.

### 2.2 Construction of adenovirus expressing shRNA targeting PRR

We also commissioned GenePharma (Shanghai, China), to construct a short hairpin RNA (shRNA) fragment targeting rat PRR, which was then introduced into recombinant adenovirus to form an adenovirus-coated PRR shRNA fragment (Ad-PRR-shRNA). The specific sequence was referred to our previous study (Xiong et al., 2019). Adenovirus-wrapped Scramble-shRNA (Ad-SC-shRNA) was also constructed as a negative control.

### 2.3 Animal models

A total of 120 eight-week-old male Wistar rats with a body weight of 200 ± 10 g were purchased from the Experimental Animal Center of Shandong University. They were fed for 1 week in an environment of alternating 12-h light/12-h dark cycle, room temperature of 20°C ± 4°C and humidity of 55% ± 5%, and then the follow-up experiments were carried out.

**FIGURE 1:**
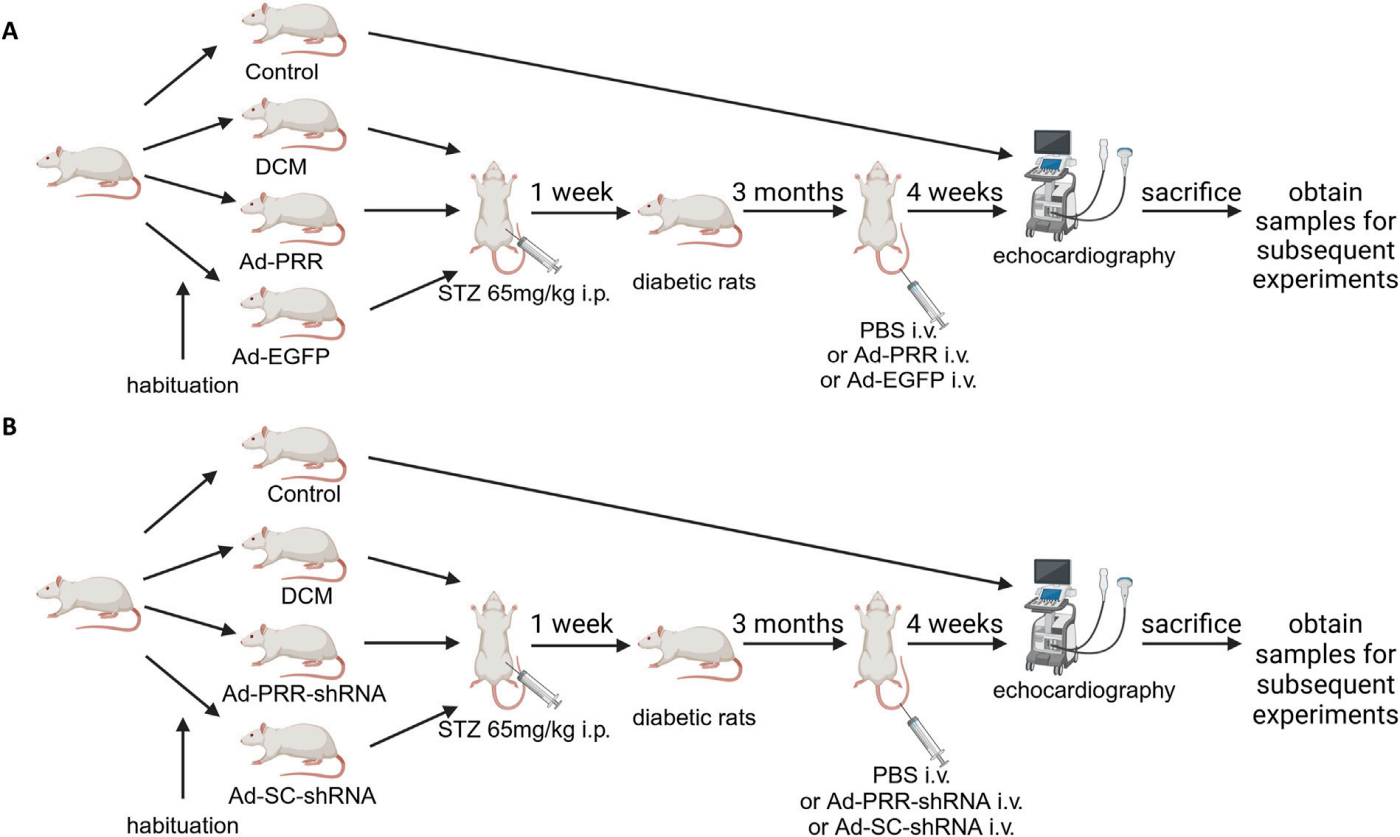
A flow chart of groups of animals and various treatments. **(A)** The flow chart of groups of animals and various treatments in the PRR overexpression experiment. **(B)** The flow chart of groups of animals and various treatments in the PRR silencing experiment.

Sixty rats were randomly selected for PRR overexpression experiment, then randomly divided into 4 groups: Control group, DCM group, Ad-PRR group, Ad-EGFP group, among which DCM group, Ad-PRR group and Ad-EGFP group were all given a single intraperitoneal high-dose injection of streptozotocin (STZ) (65 mg/kg) after 12 h fasting. After 1 week, the rats with random blood glucose >11.1 mmol/L and typical symptoms of diabetes such as polydipsia, polyuria and polydipsia were considered as successful in diabetes modeling. After 3 months of continue feeding, the DCM group, Ad-PRR group, and Ad-EGFP group were injected with phosphate-buffered saline (PBS), Ad-PRR (1 × 10^9^ pfu/200 μL, dissolved in PBS), Ad-EGFP (1 × 10^9^ pfu/200 μL, dissolved in PBS) through tail vein, respectively (Figure 1A). Two weeks after virus injection, 3 rats in each group were randomly selected to detect PRR overexpression efficiency of virus transfection.

Sixty rats were randomly selected for PRR silencing experiment, and randomly divided into Control group, DCM group, Ad-PRR-shRNA group, Ad-SC-shRNA group. DCM group, Ad-PRR-shRNA group, and Ad-SC-shRNA group were constructed diabetes model as the above PRR overexpression experiment. After feeding for 3 months, PBS, Ad-PRR-shRNA (1 × 10^9^ pfu) and Ad-SC-shRNA (1 × 10^9^ pfu) were injected through tail vein, respectively (Figure 1B).

Whether it was PRR overexpression or PRR silencing experiments, 4 weeks after the virus injection, all rats were euthanized with an intraperitoneal injection of pentobarbital (60 mg/kg) and sacrificed to obtain samples for subsequent experiments. All animal care and experimental programs were in line with the regulations on Animal Management of the Ministry of Health of the people’s Republic of China (document No. 55 of 2001). All animal experiments were approved by the Animal Protection Committee of Shandong University and the Helsinki Declaration.

### 2.4 Cell culture

The primary cardiomyocytes were rinsed with precooled PBS from the hearts of 0-3-day-old Wistar rats, then cut into pieces and digested with collagenase, the supernatant was collected and neutralized with the same amount of cardiomyocyte medium, which containing 5.5 mM glucose endotoxin free Dulbecco’s modified Eagle’s medium (DMEM, Gibco) supplemented with 10% fetal bovine serum (FBS, Gibco) and penicillin (Solarbio, 100 IU/mL). The supernatant was centrifuged at 800 rpm at 25°C for 5 min. The precipitates containing cardiomyocytes and fibroblasts were obtained and resuspended with cardiomyocyte medium. The cardiomyocytes and fibroblasts were separated according to their different adherent speeds. The resulting cardiomyocyte suspension was inoculated into a six-well plate for further experiment.

Firstly, to determine the effect of high glucose stimulation on PRR expression, DMEM (Gibco) containing 25 mM glucose and 10% FBS was used as the high glucose stimulation group (HG group), and medium containing 19.5 mM mannitol and 5.5 mM glucose was used as the high permeability control group (HP group). Secondly, to test the effect of PRR on cardiomyocyte pyroptosis under high glucose stimulation, the extracted primary cardiomyocytes were divided into two parts: PRR overexpression, PRR silencing. The former included Control group, HG group, Ad-PRR group, and Ad-EGFP group. The latter included Control group, HG group, Ad-PRR-shRNA group, and Ad-SC-shRNA group. Among them, the Ad-PRR group and the Ad-EGFP group were transfected with Ad-PRR and Ad-EGFP at 150 multiplicities of infection (MOI), respectively, and then given high glucose stimulation. The Ad-PRR-shRNA group and Ad-SC-shRNA group were transfected with Ad-PRR-shRNA and Ad-SC-shRNA (MOI = 150), respectively, and then given high glucose stimulation too. Thirdly, to validate the AMPK/NLRP3 pathway, four groups were divided: Ad-PRR group, Ad-PRR + GSK621(AMPK agonist, 30 μM, S7898, Selleck, United States) group, Ad-EGFP group, and Ad-EGFP + GSK621 group. The Ad-PRR group, Ad-PRR + GSK621 group, Ad-EGFP group and Ad-EGFP + GSK621 group were transfected with Ad-PRR or Ad-EGFP respectively, and then GSK621 was given, as shown in our previous study (Xiong et al., 2021).

### 2.5 Western blot

The protein from left ventricle of DCM rats and the treated cells were extracted by RIPA lysis buffer (Beyotime, P0013B, China) containing 1% PMSF (Solarbio, P0100, China) and 1% phosphatase inhibitors (Solarbio, P1260, China). The protein of equal amount (15–25 µg) was separated by 10% SDS-PAGE, then transferred to PVDF membrane (Millipore, United States), blocked with 5% skimmed milk for 1.5 h at room temperature, and then incubated with the diluted primary antibody against β-tubulin (1:1,000, Proteintech, 10068-1-AP, United States), NLRP3 (1:1,000, Proteintech, 10068-1-AP, United States), t-AMPK (1:1,000, Affinity, AF6423, United States), p-AMPK (1:1,000, Affinity, AF3423, United States), Caspase-1 (1:1,000, Proteintech, 22915-1-AP, United States), PRR (1:1,000, Abcam, ab40790, United Kingdom), IL-1β (1:1,000, Affinity, AF5103, United States), IL-18 (1:1,000, Proteintech, 10663-1-AP, United States) respectively, and overnight at 4°C. After washing the membrane with TBST three times, the membrane was incubated with HRP-conjugated secondary antibody (1:10,000, Proteintech, SA00001-2, United States) for 1 h at room temperature. The expression of protein was analyzed by ImageJ software.

### 2.6 Real-time PCR

Total RNA was extracted from cultured primary cardiomyocyte by using RNA Total Extraction Kit (Fastagen, 220011, China) according to the manufacturer’s instructions. NanoDrop One instrument was used to detect the concentration and purity of RNA, and the RNA was reversely transcribed into cDNA by reverse transcription kit (Takara, RR047A, Japan), and then Real-Time PCR reaction was performed by BYBR Green (Takara, RR820A, Japan). The expression of target gene was calculated by 2^−△△^CT method.

### 2.7 Histopathology and immunohistochemistry staining

At the end of the experiment, the left ventricular tissue of DCM was fixed with 4% paraformaldehyde for 24 h, washed with running water, embedded with paraffin, and cut into 4.5 µm for the subsequent experiments. HE staining and Masson staining were used to observe the cross-sectional area of cardiomyocytes and interstitial fibrosis in DCM myocardial tissue. Immunohistochemical staining was performed after dewaxing, antigen retrieval, organization background closed, and endogenous peroxidase inactivation, primary antibodies against PRR (1:200, Abcam, ab40790, United Kingdom), NLRP3 (1:200, Proteintech, 10068-1-AP, United States), Caspase-1 (1:200, Proteintech, 22915-1-AP, United States), IL-1β (1:100, Affinity, AF5103, United States), IL-18 (1:200, Proteintech, 10663-1-AP, United States), were incubated overnight at 4°C, respectively. The HRP conjugated secondary antibodies were incubated and DAB staining was performed according to the manufacturer’s instructions (ZSGB-Bio, PV-9000, ZLI-9031, China). Finally, a confocal FV1000 SPD laser scanning microscope (Olympus, Tokyo, Japan) was used to observe and photograph was taken. All the staining results were analyzed by Image Pro software (Image-Pro Plus 6.0; Media Cybernetics).

### 2.8 Immunofluorescence

The slides with treated cells of each group were washed with PBS for 3 times, fixed with 4% paraformaldehyde at room temperature fo 10 min, then washed with PBS for 3 times, then blocked with 10% bovine serum albumin (BSA, Solarbio, A8010, China) for 1 h, and incubated with PBS diluted anti-PRR (1:250, ab40790, Abcam, United Kingdom), at 4°C overnight. The PBS diluted Alexa Fluor 647 Goat Anti-Rabbit Secondary antibody (1:200, Abcam, Ab150079, United Kingdom) were incubated at room temperature for 1 h and the nucleuses were stained with DAPI (Abcam, ab104139, United Kingdom). Fluorescence images were observed under a fluorescence microscope (Leica, Wetzlar, Germany).

### 2.9 ELISA

The content of IL-1β and IL-18 in the collected cell supernatant and serum of animals in each group were tested by IL-1β ELISA kit (Boster, EK0393, China) and IL-18 ELISA kit (Boster, EK0592, China) respectively, respectively.

### 2.10 Echocardiography

After the end of the experiment, the VEVO 770 echocardiography system (Visual Sonics, Toronto, Canada) was used to examine rats in each group after anesthesia and hair removal. Mainly collected M-type signals and the left ventricular ejection fraction (LVEF), the ratio of flow Doppler E wave to A wave amplitude (E/A), left ventricular end-diastolic diameter (LVEDD), and left ventricular end-systolic diameter (LVESD) were measured.

### 2.11 Data analyses

GraphPad Prism 8.0 software (GraphPad Prism; San Diego, California, United States) was used to analyze the data. All experiments were repeated at least three times. The significance of the differences between groups was analyzed by one-way ANOVA followed by Dunnett’s multiple comparisons test. *P* < 0.05 was considered significant.

## 3 Results

### 3.1 Pyroptosis in diabetic cardiomyopathy is aggravated by the overexpression of PRR

**FIGURE 2:**
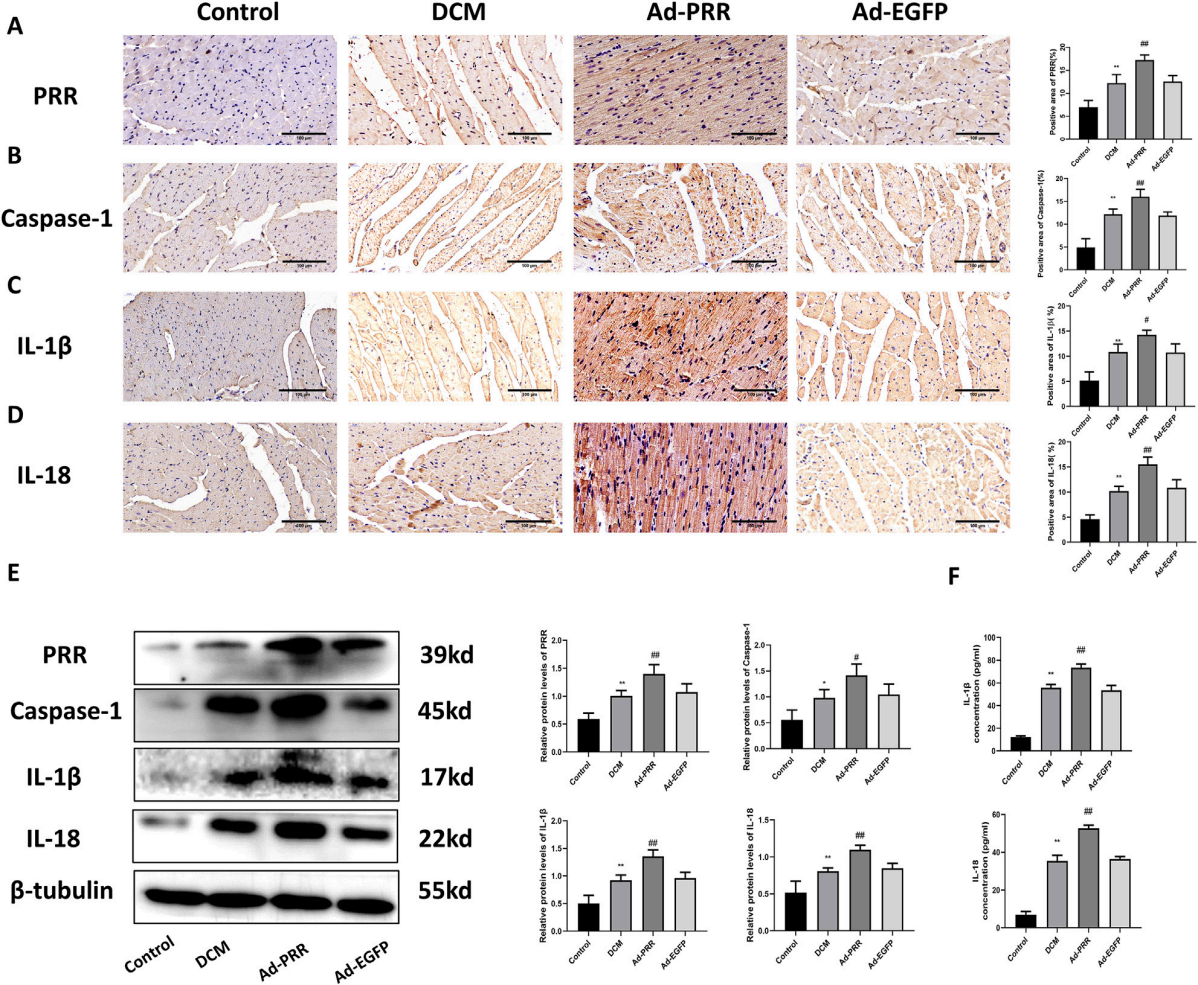
Pyroptosis in diabetic cardiomyopathy is aggravated by the overexpression of PRR. **(A–D)** Immunohistochemistry staining was used to detect the level of PRR, Caspase-1, IL-1β, IL-18 and corresponding quantitative analysis. Scale bar: 100 μm. **(E)** Western blot was used to detect the level of PRR, Caspase-1, IL-1β, IL-18 and corresponding quantitative analysis. **(F)** The level of IL-1β and IL-18 in serum were detected by ELISA. * *P* < 0.05, **P < 0.01, compared with the Control group; ^#^ *P* < 0.05, ^##^ *P* < 0.01, compared with the Ad-EGFP group.

First, we observed the expression of PRR in heart tissue from four groups. Immunohistochemical staining showed that compared with Control group, PRR expression was increased in the DCM group, and the expression level of PRR in the Ad-PRR group was significantly higher than that in the Ad-EGFP group, but there was no statistical significance between the DCM group and Ad-EGFP group (Figure 2A). This is consistent with our previous research (Xiong et al., 2021). This indicates that the intervention of PRR overexpression is successful. Next, immunohistochemical staining was also used to evaluate the level of pyroptosis in each group. The results showed that the expressions of Caspase-1, IL-1β, IL-18 in the DCM group were higher than those in the Control group, and these proteins in the Ad-PRR group were significantly higher than those in the Ad-EGFP group, but there was also no statistical significance between the DCM group and Ad-EGFP group (Figures 2B–D). Western blot was also used to detect the expression levels of PRR, Caspase-1, IL-1β, IL-18 in the myocardial tissue of DCM. The results showed that the expression level of PRR, Caspase-1, IL-1β, IL-18 in Ad-PRR group were significantly higher than the Ad-EGFP group (Figure 2E), which was consistent with the immunohistochemical staining results. In addition, we detected the level of IL-1β, IL-18 in serum by ELISA, and the results were consistent with those found in myocardial tissue (Figure 2F). These suggested that overexpression of PRR in the DCM model can significantly aggravate the level of myocardial pyroptosis in DCM.

### 3.2 The overexpression of PRR increases the activation of NLRP3 inflammasome, and decreases the phosphorylation of AMPK in DCM

**FIGURE 3:**
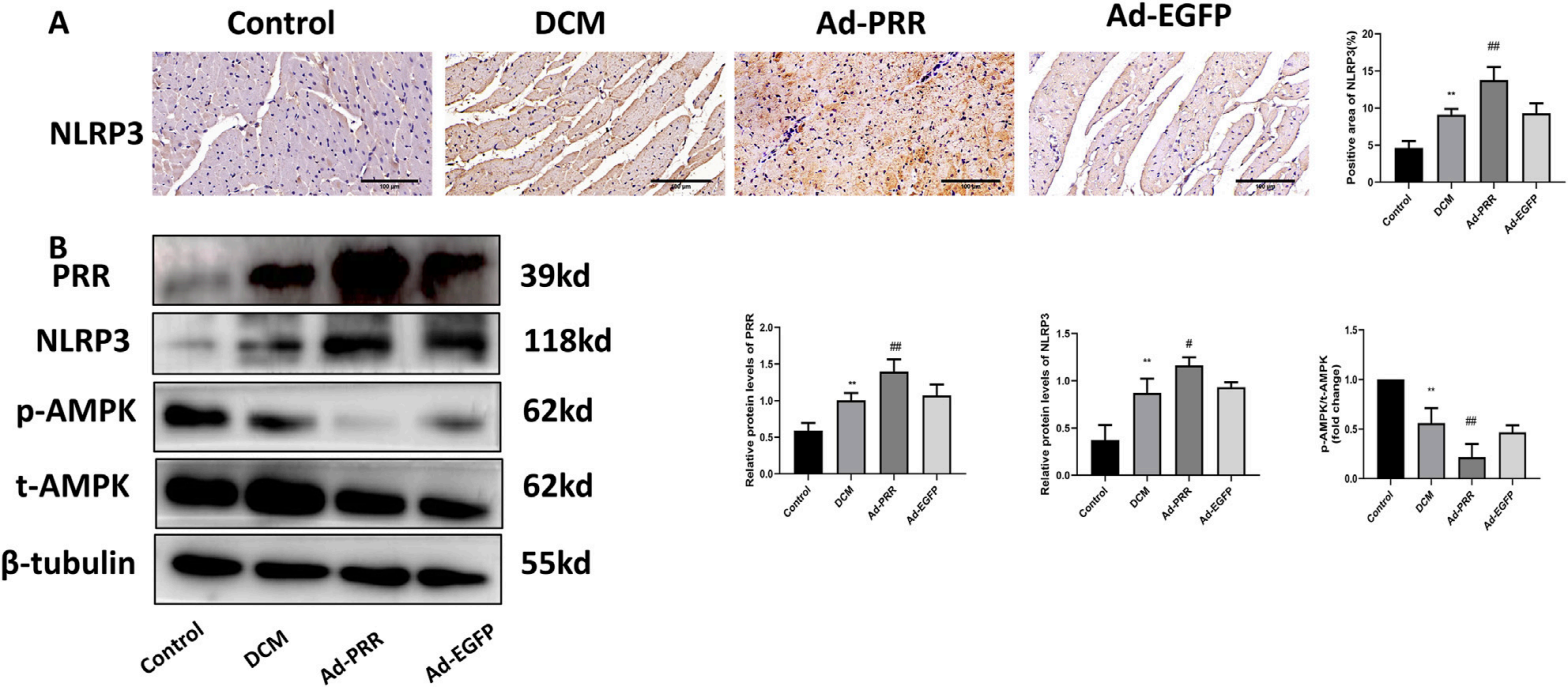
The overexpression of PRR increases the activation of NLRP3 inflammasome, and decreases the phosphorylation of AMPK in DCM. **(A)** The level of NLRP3 in myocardial tissue was detected by immunohistochemistry staining and corresponding quantitative analysis. Scale bar: 100 μm. **(B)** The level of PRR, NLRP3, p-AMPK, t-AMPK in myocardial tissue were detected by Western blot and corresponding quantitative analysis. *P < 0.05, **P < 0.01, compared with the Control group; ^#^ *P* < 0.05, ^##^ *P* < 0.01, compared with the Ad-EGFP group.

Since NLRP3 is the core molecule of pyroptosis, we detected the level of NLRP3 in myocardial tissue by immunohistochemistry staining. The result showed that the expression of NLRP3 was significantly increased in the Ad-PRR group (Figure 3A), which was consistent with the results of Western blot (Figure 3B). In addition, we also found that the phosphorylation of AMPK decreased in the DCM group and Ad-EGFP group and was even lower in the Ad-PRR group, but there was no statistical significance between the DCM group and Ad-EGFP group (Figure 3B). This suggests that overexpression of PRR in DCM model can reduce the of phosphorylation AMPK, increase the activation of NLRP3 and then aggravate the pyroptosis level in the DCM myocardium.

### 3.3 PRR overexpression aggravates the levels of fibrosis in myocardial tissue in DCM

**FIGURE 4:**
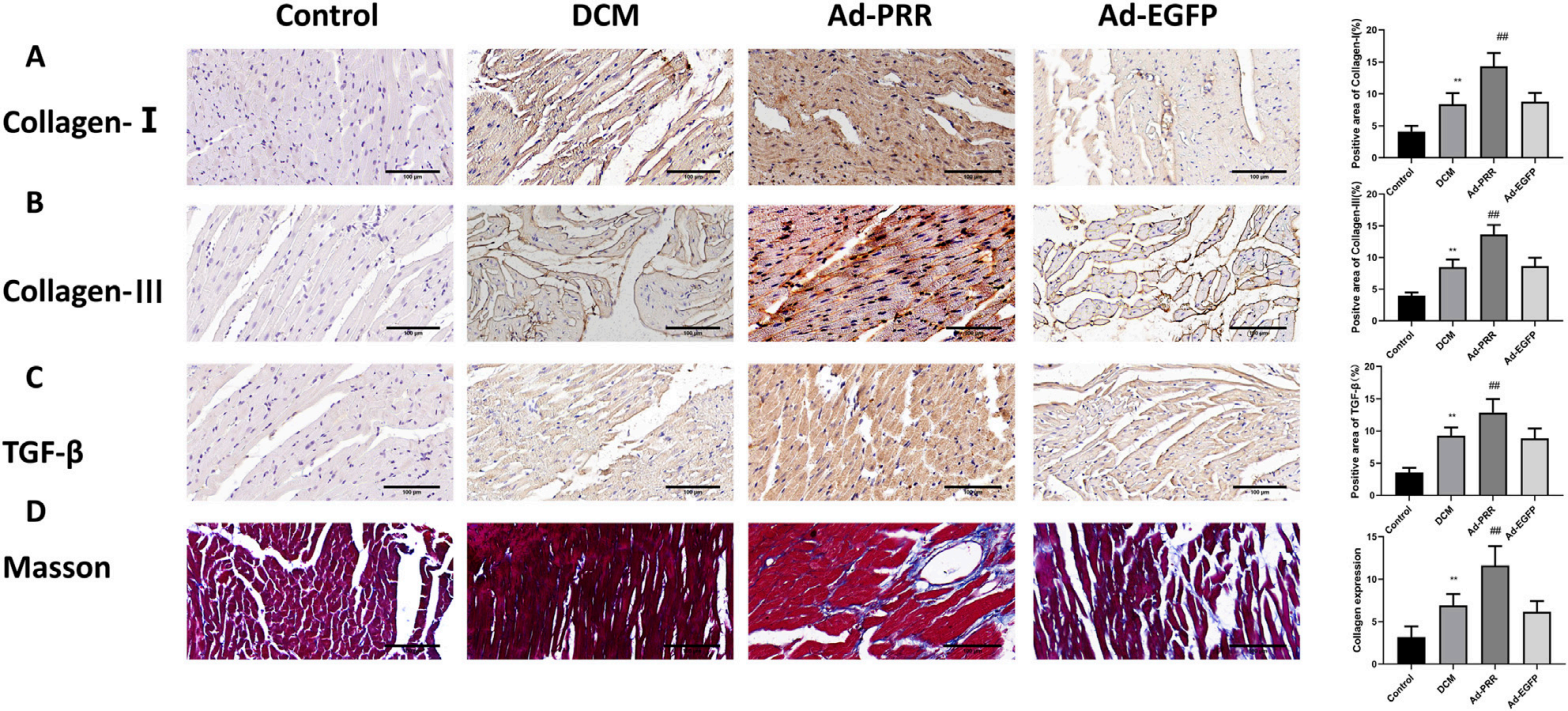
PRR overexpression aggravates the levels of fibrosis in myocardial tissue in DCM. **(A–C)** The immunohistochemical staining was used to assess the level of Collagen-Ⅰ, Collagen-Ⅲ and TGF-β in myocardial tissue. Scale bar: 100 μm. **(D)** The Masson staining was used to observe the level of collagen fiber of rats. *P < 0.05, **P < 0.01, compared with the Control group; ^#^ *P* < 0.05, ^##^ *P* < 0.01, compared with the Ad-EGFP group.

For the pathological changes of DCM, we observed the morphological changes of DCM by immunohistochemical staining and Masson staining. Immunohistochemical staining showed that the expression levels of collagen I, collagen III and TGF-β in the DCM group were higher than those in the Control group, while the expression levels of these proteins in the Ad-PRR group were higher than those in the Ad-EGFP group, but there was no statistical significance between the DCM group and Ad-EGFP group (Figures 4A–C). Masson staining results also showed that the level of collagen fiber in the DCM group was higher than that in the Control group, and that in the Ad-PRR group was higher than that in the Ad-EGFP group (Figure 4D), suggesting that the overexpression of PRR significantly aggravated the fibrosis level in the DCM myocardium.

### 3.4 The overexpression of PRR worsens cardiac function of DCM rats

**FIGURE 5:**
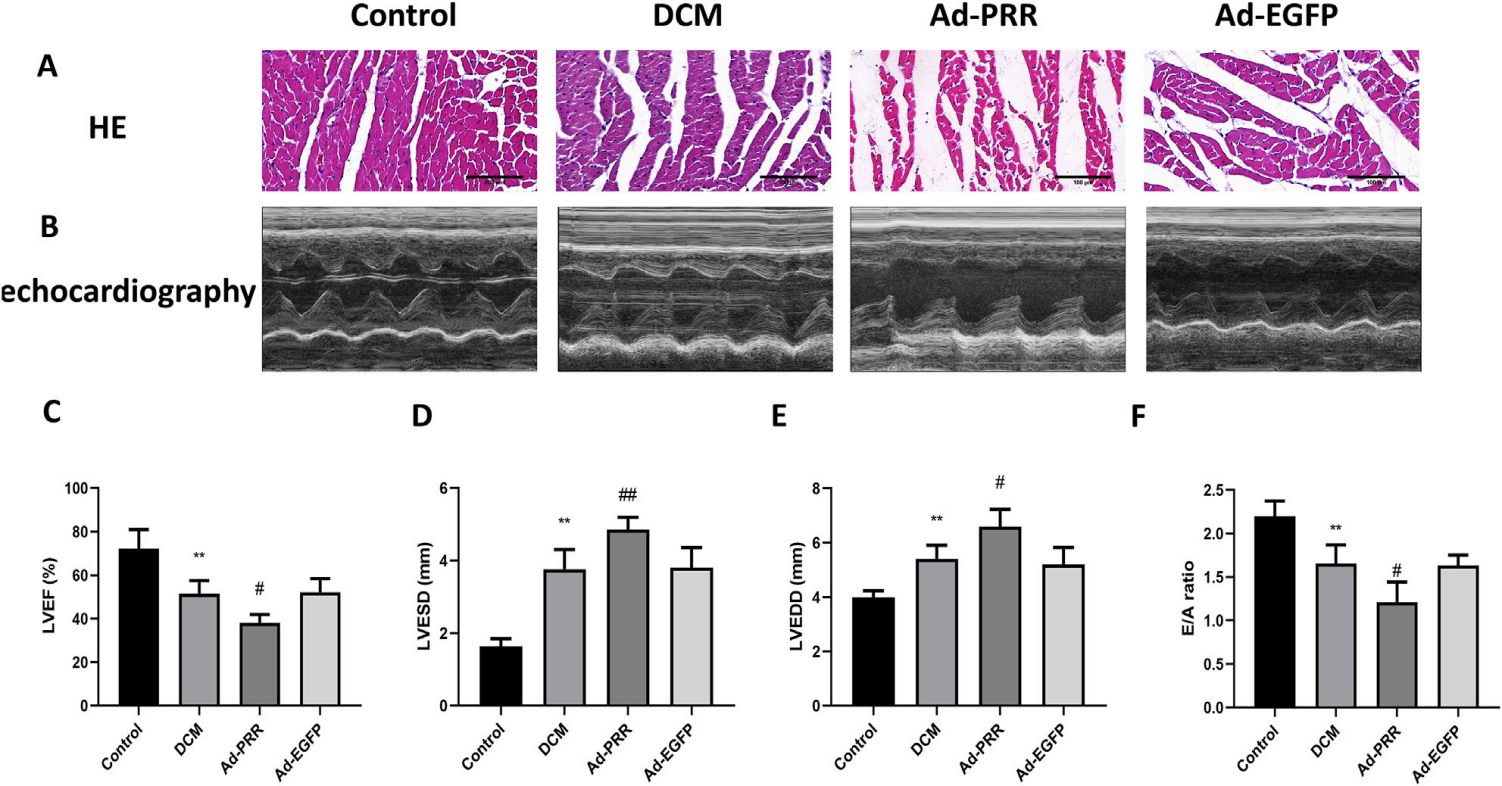
The overexpression of PRR worsens cardiac function of DCM rats. **(A)** The HE staining was used to observe the morphological changes of myocardial tissue. Scale bar: 100 μm. **(B)** The echocardiography was used to detect the change of cardiac function of rats. **(C–F)** Quantitative analysis of LVEF, LVESD, LVEDD, E/A ratio in rats. *P < 0.05, **P < 0.01, compared with the Control group; ^#^ *P* < 0.05, ^##^ *P* < 0.01, compared with the Ad-EGFP group.

HE staining was used to observe the morphological changes of myocardial tissue. The results showed the arrangement of myocardial tissue in the DCM group was sparse and slightly disordered compared with Control group, and after the overexpression of PRR, the arrangement of myocardial tissue was more disordered, but there was no statistical significance between the DCM group and Ad-EGFP group (Figure 5A).

We performed echocardiography on each group. The results showed that left ventricular ejection fraction (LVEF) of the DCM group was lower than Control group, and the LVEF of the Ad-PRR group was decreased compared with the Ad-EGFP group (Figures 5B, C). The left ventricular end diastolic diameter (LVEDD) and left ventricular end systolic diameter (LVESD) of the DCM group was larger than the Control group, and overexpressing PRR group accelerated this change (Figures 5B, D, E). In addition, we also measured the doppler E/A amplitude ratio (E/A value) of four groups. The results showed that E/A value in DCM group were decreased than Control group, and the E/A value of Ad-PRR group was significantly lower than Ad-EGFP group (Figure 5F).

### 3.5 PRR silencing reduces heart damage in DCM rats

**FIGURE 6:**
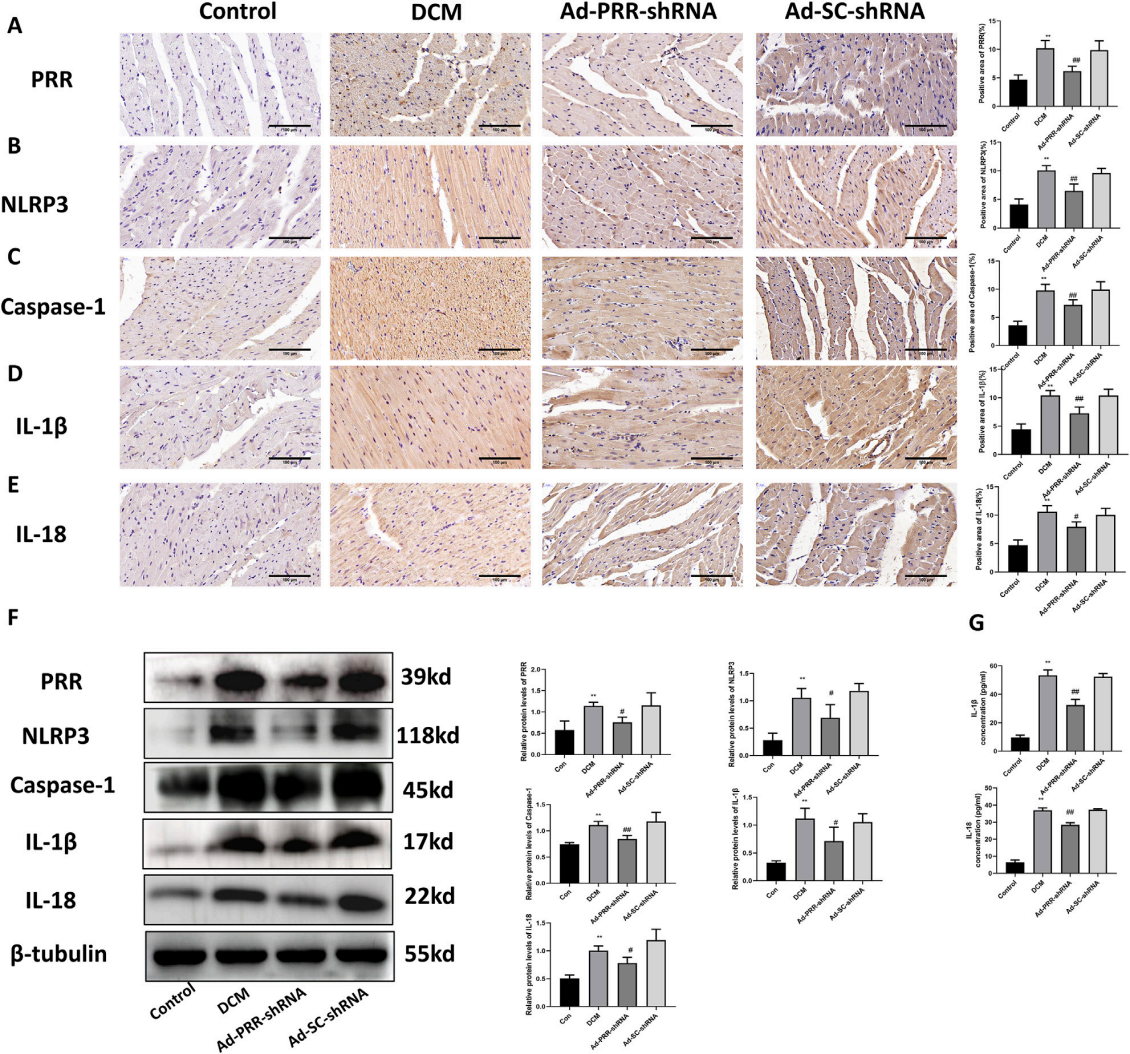
Pyroptosis in diabetic cardiomyopathy is reduced by the silencing of PRR. **(A–E)** Immunohistochemistry staining was used to detect the level of PRR, NLRP3, Caspase-1, IL-1β, IL-18 and corresponding quantitative analysis. Scale bar: 100 μm. **(F)** Western blot was used to detect the level of PRR, NLRP3, Caspase-1, IL-1β, IL-18 and corresponding quantitative analysis. **(G)** The level of IL-1β and IL-18 in serum were detected by ELISA. *P < 0.05, **P < 0.01, compared with the Control group; ^#^ *P* < 0.05, ^##^ *P* < 0.01, compared with the Ad-SC-shRNA group.

To further observe the effect of PRR on the development of DCM, we used adenovirus vector to silence the expression of PRR. We detected the level of pyroptosis in DCM rats, the immunohistochemical staining results showed that PRR silencing reduced the activation of NLRP3, and reduced the expression of Caspase-1, IL-1β, IL-18 (Figures 6A–E). This was consistent with Western blot results (Figure 6F), which means PRR silencing reduced the level of pyroptosis in DCM heart tissue. In addition, we measured the content of IL-1β and IL-18 in serum, the results indicated that PRR knock down reduced the level of IL-1β and IL-18 compared with the Ad-SC-shRNA group, but there was no significant difference between DCM group and Ad-SC-shRNA group (Figure 6G).

**FIGURE 7:**
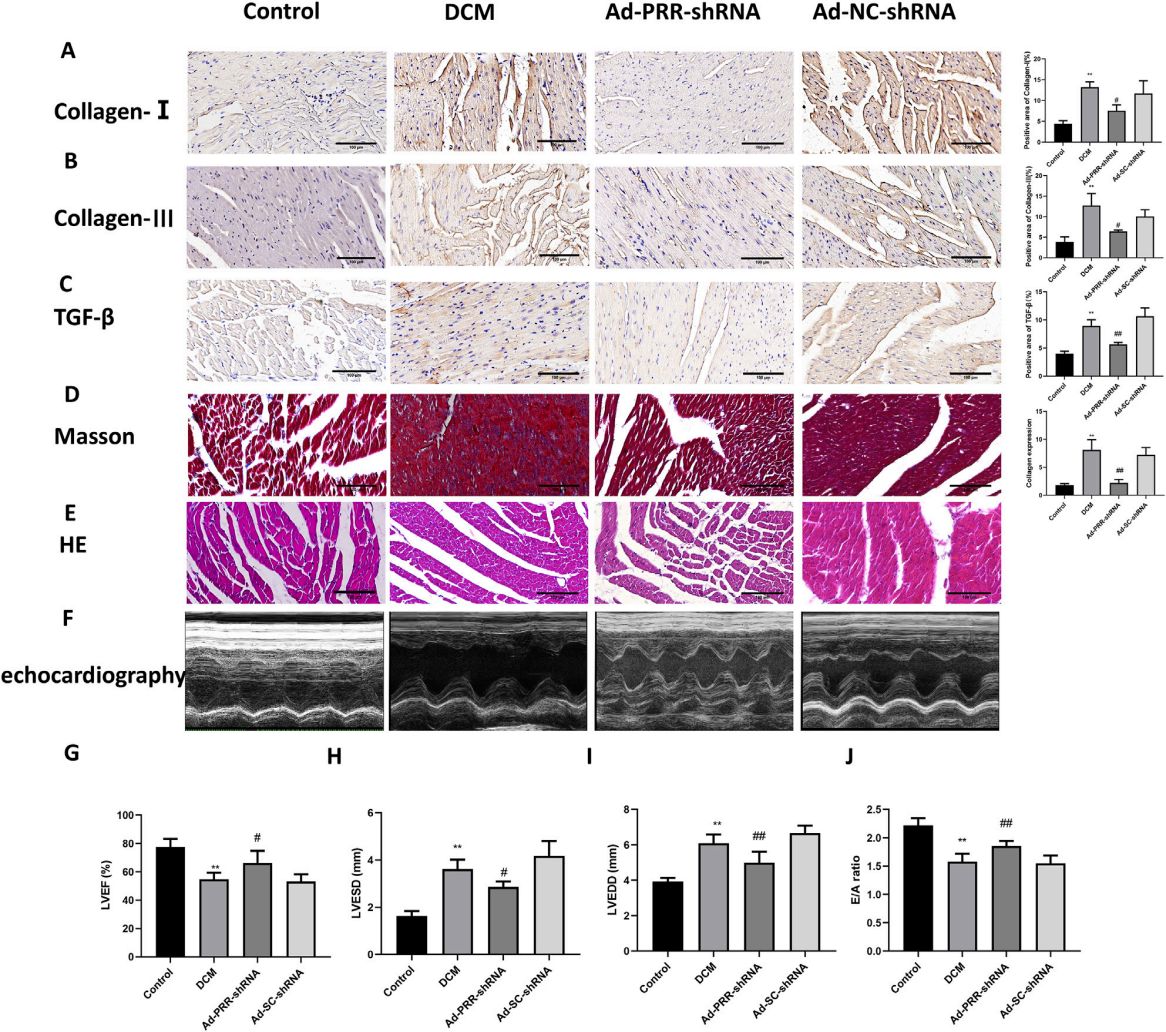
The silencing of PRR reduces heart damage in DCM rats. **(A–C)** Immunohistochemistry staining was used to detect the level of Collagen I, Collagen III, TGF-β and corresponding quantitative analysis. Scale bar: 100 μm. **(D)** The Masson staining was used to measure the deposition of collagen fiber of rats and corresponding quantitative analysis. Scale bar: 100 μm. **(E)** The HE staining was used to observe the morphological changes of myocardial tissue. Scale bar: 100 μm. **(F)** The echocardiography was used to detect the change of cardiac function of rats. **(G–J)** Quantitative analysis of LVEF, LVESD, LVEDD, E/A ratio in rats. *P < 0.05, **P < 0.01, compared with the Control group; ^#^ *P* < 0.05, ^##^ *P* < 0.01, compared with the Ad-SC-shRNA group.

Our observation also showed that the levels of collagen I, collagen III and TGF-β in the Ad-PRR-shRNA group were significantly lower than those in the Ad-SC-shRNA group, which was consistent with Masson staining results (Figures 7A–D). These indicated PRR silencing reduced fibrosis in DCM rats. HE staining also indicated that PRR silencing alleviated the disorder of myocardial alignment (Figure 7E). Next, we measured heart function of DCM rats, and found a significant improvement of LVEF and E/A ratio and decrease of LVESD and LVEDD in the Ad-PRR-shRNA group compared the Ad-SC-shRNA group, while no significant change was observed in the DCM group compared with the Ad-SC-shRNA group (Figures 7F–J). Taken together, our results indicated that PRR silencing has the potential to reduce heart damage in DCM rats.

### 3.6 High glucose stimulates the elevation of PRR expression in primary cardiomyocyte

**FIGURE 8:**
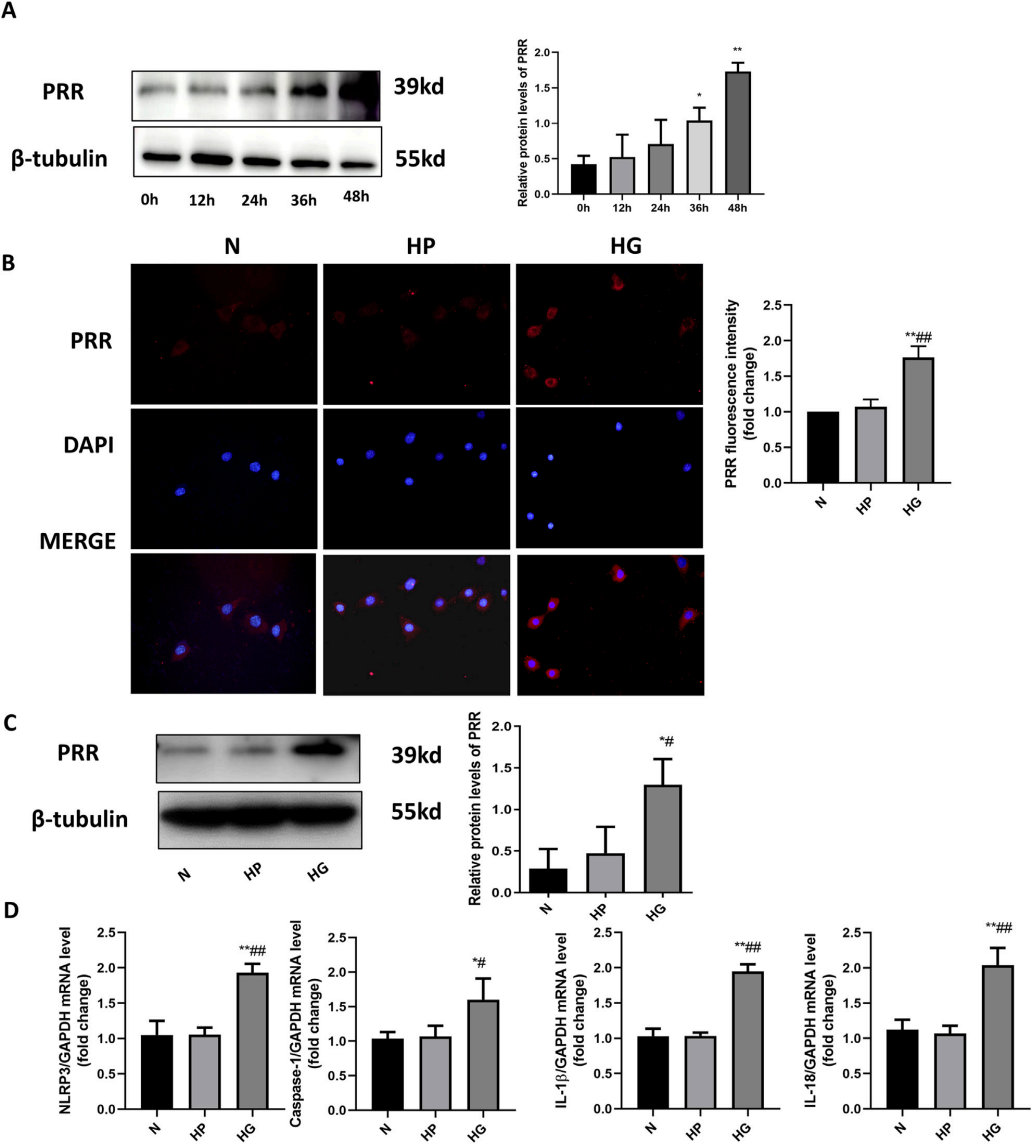
High glucose stimulates the elevation of PRR expression in primary cardiomyocyte. **(A)** Western blot was used to detect the expression of PRR under the high glucose stimulation after 0, 12, 24, 36, 48 h and the corresponding quantitative analysis. **(B)** The immunofluorescence was used to observe the expression of PRR in three groups and corresponding quantitative analysis. **(C)** Western blot was used to assess the level of PRR after 48 h of high glucose stimulation. **(D)** Real-Time PCR was used to assess the mRNA level of NLRP3, Caspase-1, IL-1β and IL-18 after 48 h of high glucose stimulation. *P < 0.05, **P < 0.01, compared with the Control group; ^#^ *P* < 0.05, ^##^ *P* < 0.01, compared with the HP group.

In order to investigate the effect of high glucose on cardiomyocytes *in vitro*, we isolated primary cardiomyocytes and set a high glucose stimulation time gradient of 0, 12, 24, 36 and 48 h. The Western blot results showed that 12, 24, 36 and 48 h all stimulated the increase of PRR expression, but 12 and 24 h have no statistical significance, and 36 and 48 h have statistical significance, so subsequent experiments use high glucose to stimulate for 48 h (Figure 8A). We also detected PRR expression by immunofluorescence, the results indicated that PRR in the HG group was significantly higher than that in the Control group and HP group after stimulating primary cardiomyocytes with high glucose to mimic hyperglycemia in diabetic rats (Figure 8B). These were consistent with the results of Western blot (Figure 8C). We also used Real-Time PCR to detect the mRNA level of NLRP3, Caspase-1, IL-1β and IL-18, the results indicated that high glucose stimulate the increase of NLRP3, Caspase-1, IL-1β and IL-18 mRNA expression (Figure 8D), which means the NLRP3 inflammasome was activated under the stimulation of high glucose.

### 3.7 Overexpression of PRR increases pyroptosis of primary cardiomyocyte

**FIGURE 9:**
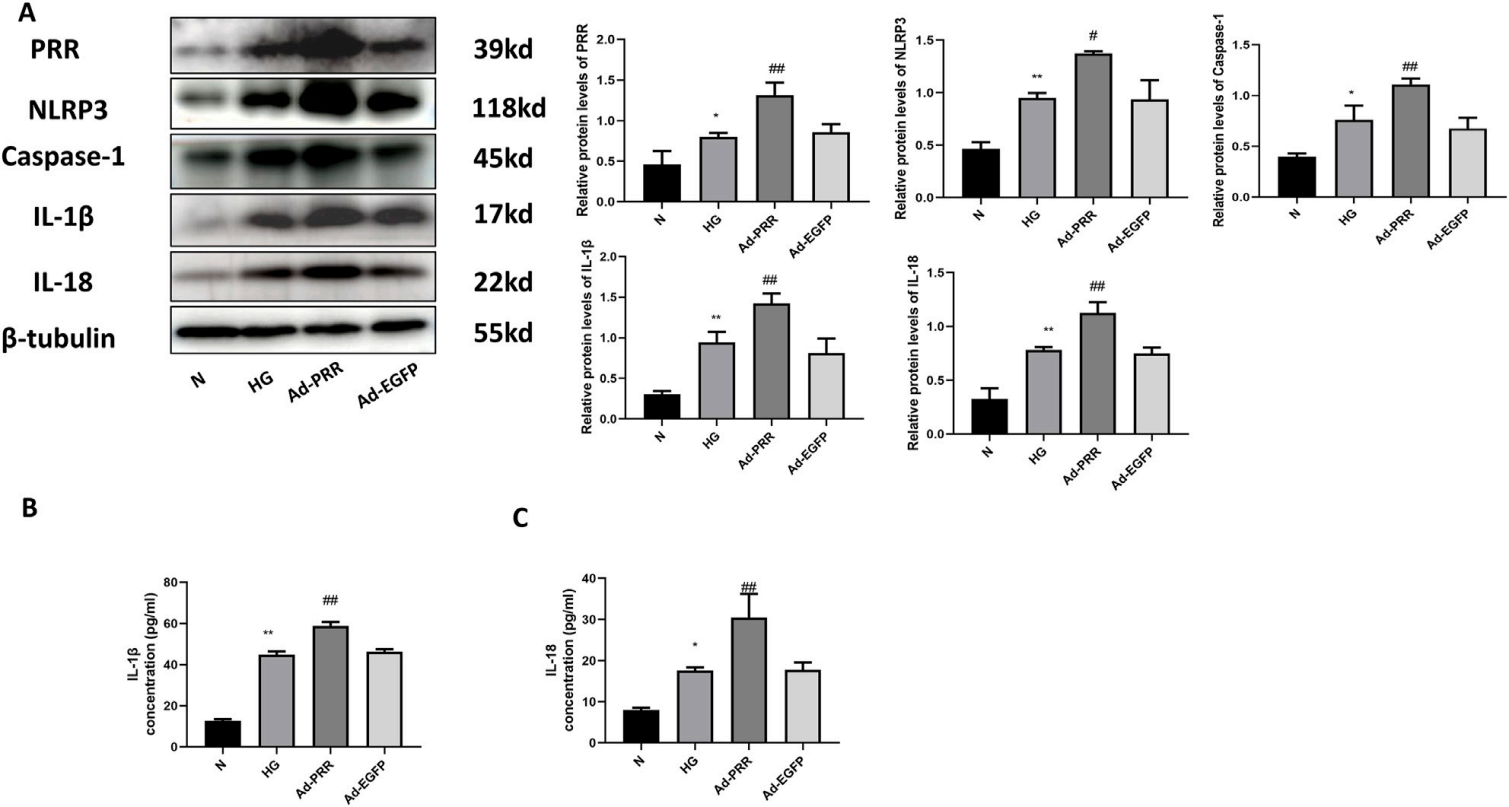
Overexpression of PRR increases pyroptosis of primary cardiomyocyte. **(A)** Western blot was used to detect the expression of PRR, NLRP3, Caspase-1, IL-1β and IL-18 and the corresponding quantitative analysis. **(B)** The ELISA was used to detect the content of IL-1β in cell culture supernatant of four groups. **(C)** The ELISA was used to detect the content of IL-18 in cell culture supernatant of four groups. *P < 0.05, **P < 0.01, compared with the Control group; ^#^ *P* < 0.05, ^##^ *P* < 0.01, compared with the Ad-EGFP group.

To further investigate the effect of PRR on pyroptosis of cardiomyocytes, we transfected the cardiomyocytes with adenovirus overexpressing PRR. Western blot results showed that the expression level of pyroptosis related proteins such as NLRP3, Caspase-1, IL-1β and IL-18 were significantly higher than that of Ad-EGFP group after overexpression of PRR (Figure 9A). We also measured the content of IL-1β and IL-18 in cell culture supernatant. The results showed that after overexpressing PRR, the content of IL-1β and IL-18 increased significantly compared with the Ad-EGFP group, but there was no significance between the HG group and Ad-EGFP group (Figures 9B, C).

### 3.8 PRR participates in the pyroptosis of primary cardiomyocyte through AMPK-NLRP3 pathway

**FIGURE 10:**
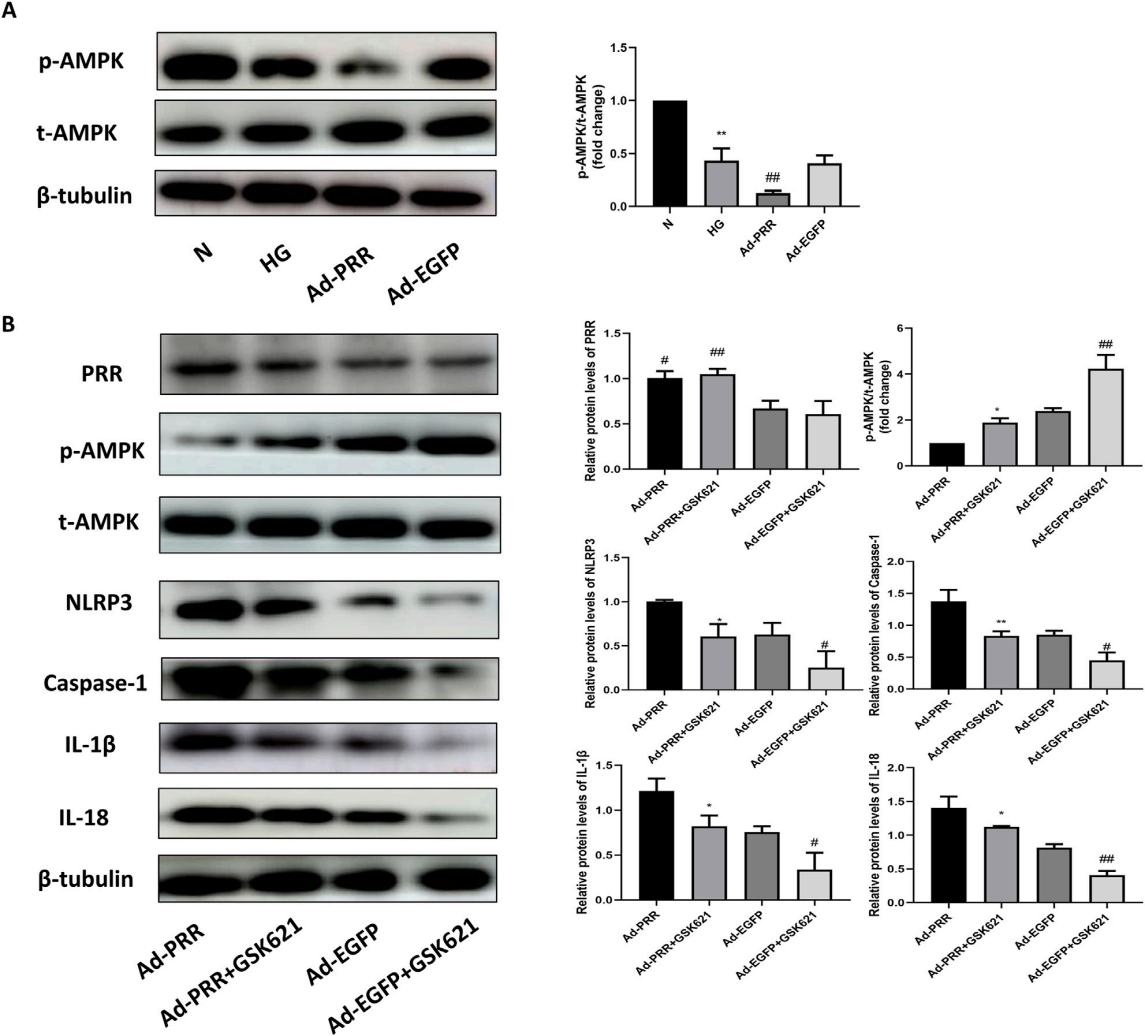
PRR participates in the pyroptosis of primary cardiomyocyte through AMPK-NLRP3 pathway. **(A)** Western blot was used to detect the expression of p-AMPK in four groups and the corresponding quantitative analysis. *P < 0.05, **P < 0.01, compared with the Control group; ^#^ *P* < 0.05, ^##^ *P* < 0.01, compared with the HG group and Ad-EGFP group. **(B)** Western blot was used to detect the expression level of p-AMPK, NLRP3, Caspase-1, IL-1β and IL-18 in four groups. *P < 0.05, **P < 0.01, compared with the Ad-PRR group; ^#^ *P* < 0.05, ^##^ *P* < 0.01, compared with the Ad-EGFP group.

In order to explore the specific mechanism of PRR regulating cardiomyocyte pyroptosis, firstly, we detected the activation level of AMPK in each group. Western blot analysis showed that the phosphorylation level of AMPK was significantly lower in the HG group than in the Control group, and was more significantly lower in the Ad-PRR group than in the Ad-EGFP group (Figure 10A). Previous studies have shown that AMPK activation can reduce NLRP3 inflammasomes in a variety of diseases, such as diabetes and ischemic stroke. To verify whether PRR regulates pyroptosis through AMPK, we administered the AMPK agonist GSK621 to each group, which can increase the phosphorylation level of AMPK. It was found that the expression level of NLRP3, Caspase-1, IL-1β and IL-18 in the Ad-PRR + GSK621 group were significantly lower than that in the Ad-PRR group alone, Ad-EGFP + GSK621 group was significantly lower than Ad-EGFP group, respectively (Figure 10B). This suggests that PRR may promote the activation of NLRP3 inflammasome by reducing AMPK phosphorylation level, thus aggravating the pyroptosis level.

### 3.9 Inhibition of PRR reduces the pyroptosis of primary cardiomyocyte under high glucose stimulation

**FIGURE 11:**
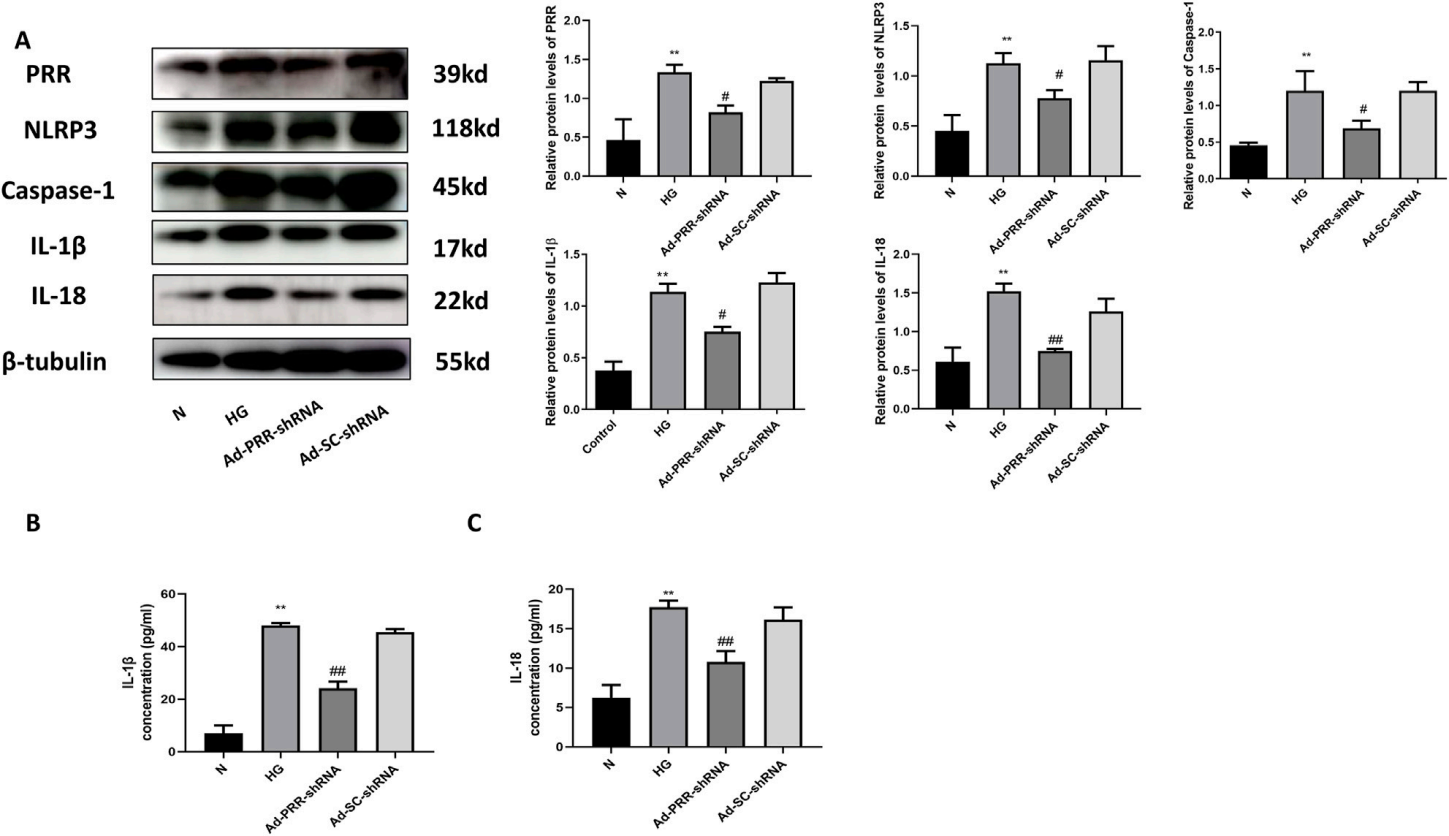
Inhibition of PRR reduces the pyroptosis of primary cardiomyocyte under high glucose stimulation. **(A)** Western blot was used to detect the expression of NLRP3, Caspase-1, IL-1β and IL-18 in four groups and the corresponding quantitative analysis. **(B)** The ELISA was used to detect the content of IL-1β in cell culture supernatant of four groups. **(C)** The ELISA was used to detect the content of IL-18 in cell culture supernatant of four groups. *P < 0.05, **P < 0.01, compared with the Control group; ^#^ *P* < 0.05, ^##^ *P* < 0.01, compared with the Ad-SC-shRNA group.

To further explain that PRR is involved in pyroptosis of primary cardiomyocyte, we transfected cardiomyocytes with adenovirus containing Ad-PRR-shRNA, and found that compared with the Ad-SC-shRNA group, the expression level of NLRP3, Caspase-1, IL-1β, IL-18 in the Ad-PRR-shRNA group were decreased (Figure 11A), suggesting that inhibition of PRR expression under high glucose stimulation could partially alleviate the pyroptosis injury. The ELISA results also showed that content of IL-1β and IL-18 in cell culture supernatant in the Ad-PRR-shRNA group were significantly decreased compared with the Ad-SC-shRNA group (Figures 11B, C), which means PRR silencing reduced the secretion of IL-1β and IL-18. This indicated PRR played an important role in myocardial pyroptosis.

## 4 Discussion

Caspase-1, IL-1β, IL-18, and the process of pyroptosis are primarily associated with immune cells such as monocytes, macrophages, and dendritic cells. However, caspase-1, IL-1β and IL-18 also have significant expression in cardiomyocytes in the context of inflammation. This suggests that while the inflammatory response primarily involves immune cells, non-immune cells, particularly in tissues like the heart, also contribute to the expression of these inflammatory mediators (Chen et al., 2021; Li et al., 2020). In our study, we focus on how does the (Pro) renin receptor enhance myocardial injury in diabetic cardiomyopathy and participate in the pyroptosis of cardiomyocytes via AMPK-NLRP3 pathway. And there are many studies reported the pathological effect of pyroptosis on cardiomyocytes. For example, Li et al. demonstrated that NLRP3 activated by STING-IRF3 is involved in lipopolysaccharide-induced cardiac dysfunction, inflammation, apoptosis, and pyroptosis, they found that STING binds to type Ⅰ interferon (IFN) regulatory factor 3 (IRF3) and phosphorylates it, thereby promoting the activation of NLRP3 and aggravating pyroptosis of sepsis-induced cardiomyopathy (Li et al., 2019). Zeng et al. found that NLRP3 inflammasome-mediated pyroptosis was hyper activated in the myocardial tissues of dilated cardiomyopathy patients and was negatively correlated with cardiac function (Zeng et al., 2020). In this study, our main finding was that PRR overexpression aggravated myocardial pyroptosis of DCM *in vitro* and *in vivo* studies. In contract, PRR silencing inhibited myocardial pyroptosis. Furthermore, we found that PRR participated in the pyroptosis of primary cardiomyocyte through AMPK-NLRP3 pathway. Then, we speculated that PRR-AMPK-NLRP3 pathway plays a key role in the myocardial pyroptosis of DCM.

As a common and important cardiovascular complication of diabetes, diabetic cardiomyopathy (DCM) has been the main cause of heart failure in diabetic patients for the past 50 years since the concept was proposed by Rubler et al. in 1972 (Boudina and Abel, 2010). It has been confirmed that the occurrence and development of DCM has a variety of molecular mechanisms, the key mechanisms involved in DCM pathophysiology include oxidative stress, hyperglycemia and glucotoxicity, lipotoxicity, mitochondrial disfunction, autophagy changes, calcium treatment damage, excessive activation of RAS system, advanced glycation end products (AGEs) deposition, inflammation, cardiac autonomic neuropathy (CAN) and microvascular disfunction, etc (Ritchie and Abel, 2020; Tan et al., 2020; Jia et al., 2018). Although clinical research and basic research on DCM have increased exponentially in the past few decades, the pathogenesis of this disease has not been fully clarified. There is still a lack of effective clinical treatment methods. In this study, we provide evidence for PRR participating in DCM myocardial pyroptosis. We also found that PRR aggravated DCM myocardial pyroptosis through the AMPK-NLRP3 pathway. These findings provided a new understanding of the pathogenesis of DCM and a new theoretical basis for the prevention and treatment of DCM.

Recent studies have shown that the pyroptosis of cardiomyocytes is an important event in the progression of DCM disease (Cai et al., 2022; Zeng et al., 2019). In DCM, high glucose stimulated the production of reactive oxygen species and inflammation accompanied by increased expression of cleaved Caspase-1, IL-1β and IL-18 (Gan et al., 2020). Studies have shown that MMPs/TIMPs plays an important role in the pathological process of DCM, the expression of TIMP-2 gene and protein content in the myocardium significantly increases, while the expression of MMP-2 gene and protein significantly decreases. This imbalance in the MMP/TIMP ratio directly leads to insufficient degradation of extracellular matrix collagen, which manifests as extracellular matrix collagen deposition in myocardial cells (Li et al., 2007). High glucose and Ang Ⅱ can weaken the activity of MMP-2, relatively enhance the biological activity of TIMPs, and also lead to an increase in the content of type Ⅰ and type III collagen and the overall collagen in myocardial (Westermann et al., 2007). In addition, studies have shown that the activation of inflammasome and the release of inflammatory factors can lead to collagen deposition and fibrosis (Gill et al., 2023), thus accelerating the pathological progress of DCM (Fuentes-Antras et al., 2014). However, it has not been reported whether PRR is involved in cardiomyocyte pyroptosis in DCM. In this study, we found that PRR expression was elevated in the myocardial tissue of DCM group, which was consistent with our previous research results (Yu et al., 2021). Similarly, *in vitro* cardiomyocytes, we found that high glucose stimulated the expression of PRR in time-dependent. After overexpression of PRR, the level of NLRP3, Caspase-1, IL-1β, IL-18 was obviously increased. Moreover, the myocardial fibrosis level of DCM rats increased, and the cardiac function deteriorated significantly. This suggests that the overexpression of PRR aggravated the level of NLRP3 mediated pyroptosis and worsened the heart function. We further experimented with adenovirus containing Ad-PRR-shRNA and found that PRR silencing could reduce the level of pyroptosis, fibrosis and improve cardiac function of DCM rats. The same results in in vitro cell experiments have been found.

AMP-activated protein kinase (AMPK) plays a key role in the regulation of a variety of physiological and pathological processes, including energy metabolism and oxidative stress (Daskalopoulos et al., 2016). A study reported that metformin can activate AMPK chronically and prevent cardiomyopathy by restoring mitochondria and cardiac ultrastructure in OVE26 diabetic mice (Xie et al., 2011). Up to now, it is not known whether AMPK is involved in PRR-mediated cardiomyocyte pyroptosis. In this study, we found that high glucose stimulation decreased the level of AMPK phosphorylation in primary cardiomyocyte, and further decreased the level of AMPK phosphorylation in PRR overexpression group, suggesting that PRR overexpression inhibited AMPK phosphorylation. In order to verify whether AMPK was involved in this process, we used GSK621, an AMPK agonist, which increased the level of AMPK phosphorylation on the basis of PRR overexpression compared with the PRR overexpression alone. And after used GSK621, the activation of downstream NLRP3 inflammasome in primary cardiomyocyte decreased and the expression of Caspase-1, IL-1β and IL-18 reduced. This was consistent with some studies (Li et al., 2022; Luo et al., 2022; Yang et al., 2019), and further suggests that PRR regulates NLRP3-mediated pyroptosis through AMPK pathway. However, a slight deficiency is that we have only verified the role of AMPK agonist in cell experiments *in vitro*, and more experiments will be needed in the future to explore more mechanisms of PRR regulating AMPK.

In conclusion, our results strongly demonstrated that PRR aggravated cardiac injury by inhibiting AMPK and then activating NLRP3 inflammasome in DCM. Furthermore, we found that PRR-AMPK-NLRP3 pathway plays a key role in the myocardial pyroptosis of DCM. This study provided new ideas for the prevention and treatment of DCM.

## Data Availability

The original contributions presented in the study are included in the article/supplementary material, further inquiries can be directed to the corresponding authors.
