# Corrigendum: (Pro)renin receptor aggravates myocardial pyroptosis in diabetic cardiomyopathy through AMPK-NLRP3 pathway

**DOI:** 10.3389/fphar.2024.1532331

**Published:** 2024-12-13

**Authors:** Shengnan Li, Jingjing Zhang, Yuewen Zhao, Li Kang, Haipeng Jie, Bo Dong

**Affiliations:** ^1^ Department of Cardiology, Shandong Provincial Hospital, Cheeloo College of Medicine, Shandong University, Jinan, China; ^2^ National Key Laboratory for Innovation and Transformation of Luobing Theory, The Key Laboratory of Cardiovascular Remodeling and Function Research, Department of Cardiology, Chinese Ministry of Education, Chinese National Health Commission and Chinese Academy of Medical Sciences, Qilu Hospital of Shandong University, Jinan, China; ^3^ Department of Cardiology, Shandong Provincial Hospital Affiliated to Shandong First Medical University, Jinan, China; ^4^ Division of Cellular and Systems Medicine, School of Medicine, University of Dundee, Dundee, Scotland, United Kingdom

**Keywords:** (Pro)renin receptor, diabetic cardiomyopathy, pyroptosis, NLRP3, AMPK (AMP-activated protein kinase)

In the published article, there was an error in Figure 7 as published. In Figures 7G, J the analysis diagram for LVEF was erroneously excluded and an incorrect E/A analysis diagram was inserted in its place**.** The corrected Figure 7 and its caption appear below.

**FIGURE 7:**
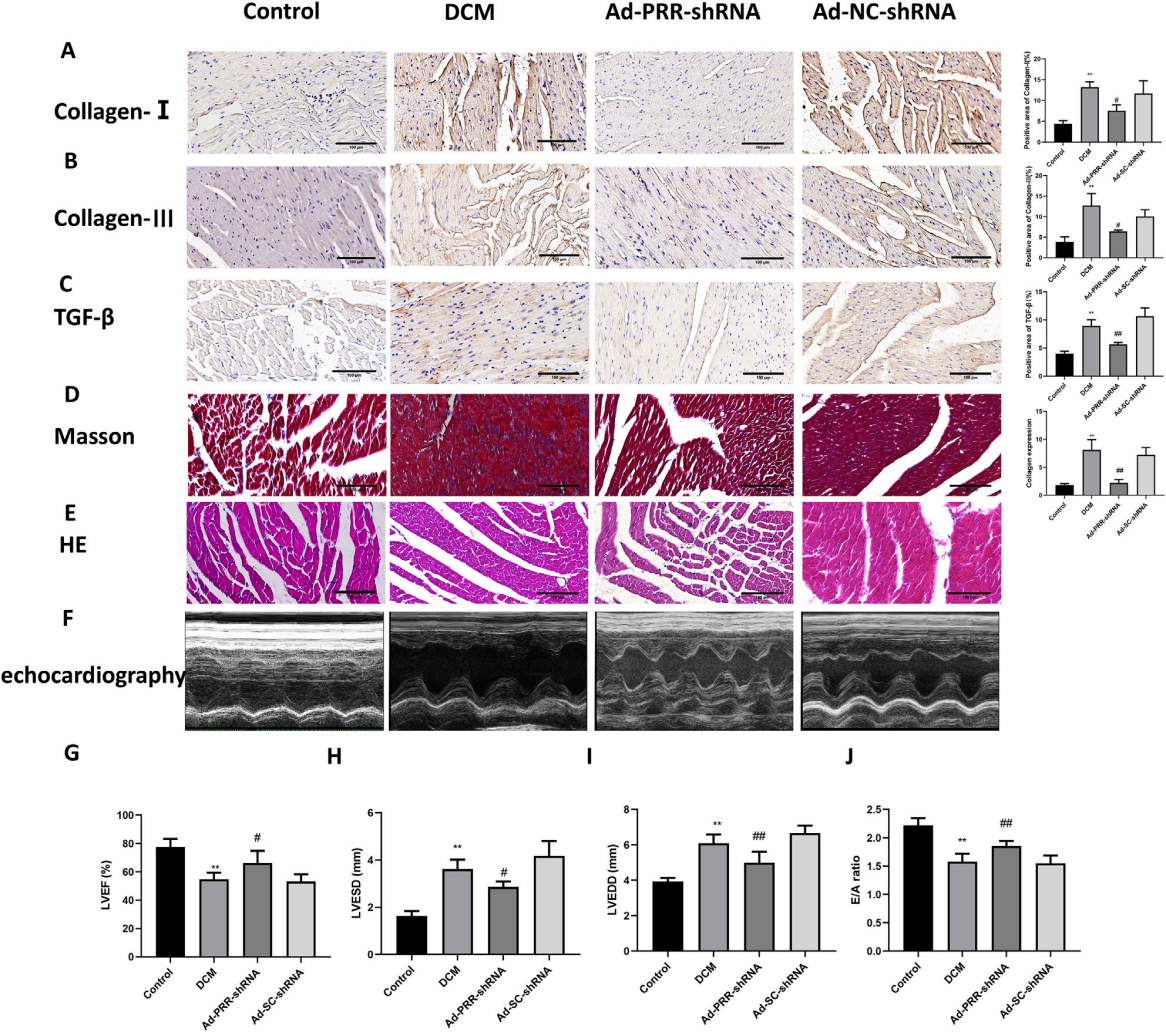
The silencing of PRR reduces heart damage in DCM rats. **(A–C)** Immunohistochemistry staining was used to detect the level of Collagen Ⅰ, Collagen Ⅲ, TGF-β and corresponding quantitative analysis. Scale bar: 100 μm. **(D)** The Masson staining was used to measure the deposition of collagen fiber of rats and corresponding quantitative analysis. Scale bar: 100 μm. **(E)** The HE staining was used to observe the morphological changes of myocardial tissue. Scale bar: 100 μm. **(F)** The echocardiography was used to detect the change of cardiac function of rats. **(G–J)** Quantitative analysis of LVEF, LVESD, LVEDD, E/A ratio in rats. *P < 0.05, **P < 0.01, compared with the Control group; ^#^ *P* < 0.05, ^##^ *P* < 0.01, compared with the Ad-SC-shRNA group.

The authors apologize for this error and state that this does not change the scientific conclusions of the article in any way. The original article has been updated.

